# Improvement of the pharmacological activity of menthol via enzymatic β-anomer-selective glycosylation

**DOI:** 10.1186/s13568-017-0468-0

**Published:** 2017-08-29

**Authors:** Ha-Young Choi, Bo-Min Kim, Abubaker M. A. Morgan, Joong Su Kim, Won-Gon Kim

**Affiliations:** 1 Superbacteria Research Center, Korea Research Institute of Bioscience and Biotechnology, Yusong, Daejeon, 305-806 Republic of Korea; 2 Department of Bio-Molecular Science, KRIBB School of Bioscience, Korea University of Science and Technology (UST), Yusong, Daejeon, Republic of Korea; 3 Infection Control Material Research Center, Korea Research Institute of Bioscience and Biotechnology, Jeongeup-si, Jeollabuk-do 56212 Republic of Korea

**Keywords:** Menthol, Menthol β-glycosides, Glycosyltransferase, Solubility, Cooling

## Abstract

Menthol has a considerable cooling effect, but the use range of menthol is limited because of its extremely low solubility in water and inherent flavor. (−)-Menthol β-glucoside was determined to be more soluble in water (>27 times) than (−)-menthol α-glucoside; hence, β-anomer-selective glucosylation of menthol is necessary. The in vitro glycosylation of (−)-menthol by uridine diphosphate glycosyltransferase (BLC) from *Bacillus licheniformis* generated (−)-menthol β-glucoside and new (−)-menthol β-galactoside and (−)-menthol *N*-acetylglucosamine. The maximum conversion rate of menthol to (−)-menthol β-d-glucoside by BLC was found to be 58.9%. Importantly, (−)-menthol β-d-glucoside had a higher cooling effect and no flavor compared with menthol. In addition, (−)-menthol β-d-glucoside was determined to be a non-sensitizer in a skin allergy test in the human cell line activation test, whereas menthol was a sensitizer.

**Electronic supplementary material:**

The online version of this article (doi:10.1186/s13568-017-0468-0) contains supplementary material, which is available to authorized users.

## Introduction

Menthol is used in a wide variety of products as an additive to foods, medicines, cosmetics, and cigarettes owing to its inherent mint flavor and refreshing feelings. The use range of menthol, however, is limited because of its extremely low solubility in water and flavor (Kamatou et al. 2013; Patel et al. 2007). Glycosylation is one method than can improve both the water solubility and biological activity of non-glycosylated compounds (Ahmed et al. 2006). Sugar moieties are known to improve the pharmacokinetic properties and/or affect the biological activity of glycosides in important natural pharmaceutical products (Weymouth-Wilson 1997). Chemical synthesis of menthyl glucosides was reported to produce an anomeric mixture of α- and β-anomers (Sakata and Iwamura 1979).

As an eco-friendly process, enzymatic synthesis is superior to chemical synthesis because the enzymatic reactions proceed regioselectively and stereoselectively without the need for a protection and deprotection processes. α-Anomer-selective glucosylation of menthol by yeast α-glucosidase has been reported (Nakagawa et al. 1998), but to the best of our knowledge, β-anomer-selective glucosylation of menthol has not yet been reported.

*Bacilli* have been used as a source of glycosyltransferases for the modification of natural products. Uridine diphosphate (UDP)-glycosyltransferases from *Bacillus* sp. HH1500 (Rabausch et al. 2013) and *B. cereus* (Ahn et al. 2009) were reported to glucosylate macrolide and flavonoid substrates. UDP-glycosyltransferase BLC from *B. licheniformis* has been known to glycosylate a variety of natural products such as geldanamycin analogs, epothilone A, resveratrol, chalcone, and various flavonoids (Koirala et al. 2014; Parajuli et al. 2014; Wu et al. 2012). Additionally, BLC was reported to catalyze β-anomer-selective glycosylation of geldanamycin analogs and epothilone A. These reports led us to study β-anomer-selective glycosylation of menthol by BLC.

Herein, we report that the water solubility of the β-anomer of menthyl glucoside is superior to its α-anomer, and *Bacillus* glycosyltransferase BLC catalyzes the β-anomer-selective glycosylation of (−)-menthol. Additionally, the cooling ability and skin sensitization properties of (−)-menthol β-d-glucoside were improved compared with those of menthol.

## Materials and methods

### Chemicals

(−)-Menthol, UDP-glucose, UDP-galactose, UDP-*N*-acetylglucosamine, and UDP-glucuronic acid were purchased from Sigma-Aldrich (St. Louis, MO, USA). The chemical synthesis of (−)-menthol β-glucoside and (−)-menthol α-glucoside was conducted by MediGen Inc (Daejeon, Korea).

### Cloning, expression, and purification of glycosyltransferase

Glycosyltransferase BLC was cloned, expressed, and purified from the genomic DNA of *B. licheniformis* DSM13, as reported previously (Wu et al. 2012). Briefly, the amplified PCR products were purified, digested with *Bam*HI and *Xho*I, and cloned into the same sites of the pET28a vector. The recombinant expression vectors were transformed into *E. coli* BL21(DE3). The *E. coli* BL21(DE3) culture transformed with the recombinant expression vector was induced with isopropyl-1-thio-β-d-galactopyranoside (IPTG) (at a final concentration of 0.5 mM). After centrifugation of the cell lysate, BLC enzyme was purified by nickel–nitriloriacetic acid (Ni–NTA) column chromatography (Qiagen). The fractions eluted with 50 and 100 mM imidazole were pooled and concentrated with an Amicon Ultra-15 instrument (Millipore). The concentrated enzyme was dialyzed overnight at 4 °C using 100 mM Tris–HCl (pH 8.0) containing 20% (vol/vol) glycerol and stored at 4 °C until use.

### Glycosylation of menthol using BLC

Analytical glycosylation reactions were performed in a total volume of 50 μL containing purified His-tagged recombinant BLC (3 μM), 1 mM menthol, 2 mM UDP-glycosides (UDP-glucose, UDP-galactose, UDP-*N*-acetylglucosamine, and UDP-glucuronic acid), 1 mM MgCl_2_, and 50 mM Tris–HCl (pH 8.0) (Wu et al. 2012). The reaction mixtures were incubated at 30 °C for 18 h. For determination of the kinetic parameters for menthol, the reactions were performed for 10 min with varying concentrations of menthol from 0.25 to 4 mM. Triplicate reactions were performed. All reactions were quenched by the addition of 450 μL MeOH and then centrifuged. The supernatants were qualitatively detected by LC–ESI–MS or quantitatively analyzed by LC–MS/MS operating in multiple reaction monitoring (MRM) mode, as described below.

### Quantitative analysis of menthol glucoside by LC–MS/MS

The concentrations of menthol glucoside in the water solubility test or the BLC reactions were analyzed by LC–MS/MS in MRM mode. The samples were subjected to an HPLC system (Luna C18(2), 100 × 2.0 mm, 3 μm, Phenomenex, Torrance, CA, USA) connected to a QTrap 3200 with a Turbolon Spray source (AB SCIEX, Singapore). The column was maintained at 20 °C with a flow rate of 0.4 mL/min and a gradient of 0.1% (v/v) formic acid in H_2_O (A) and 0.1% (v/v) formic acid in acetonitrile (B) at 40% B to 90% B for 7 min. MRM was performed by selecting the two mass ions set specifically for the selected analytes to detect the transition from parent ion to product ion, i.e., *m/z* 319 > 139 for (−)-menthol β-glucoside. For analysis of (−)-menthol β-d-glucoside, the Turbolon Spray source-dependent parameters were optimized to the following values: 10 psi curtain gas, high collision gas, 5500 V ion spray voltage, 150 °C temperature, and 12 psi ion source gas. The compound-dependent parameters were optimized to the following values: 15 eV collision energy, 8.5 V entrance potential, 126 V declustering potential, and 4 V collision cell exit potential. For analysis of (−)-menthol α-d-glucoside, the Turbolon Spray source-dependent parameters were the same as those for (−)-menthol α-d-glucoside. The compound-dependent parameters were optimized to the following values: 15 eV collision energy, 4 V entrance potential, 41 V declustering potential, and 4 V collision cell exit potential.

### Isolation and structure determination of three biosynthesized menthol glycosides

To obtain a large amount of menthol glycosides for NMR study, the preparative-scale reaction containing UDP-d-glucose (2 mM, 39.1 mg), menthol (1 mM, 5 mg), 3 μM BLC in 32 mL was performed for 18 h, producing menthol glucoside (0.41 mM, 4.2 mg) representing 41.2% conversion of menthol. The preparative-scale reaction containing UDP-d-galactose (2 mM, 78.2 mg), menthol (8 mM, 80 mg), and 3 μM BLC in 64 mL was performed for 18 h, producing of menthol galactoside (0.125 mM, 2.6 mg) representing 1.56% conversion of menthol. To purify the menthol glycosides from the reaction mixture, the reaction mixture was partitioned with water-saturated butanol, and the butanol layer was evaporated in vacuo. The resulting residue was purified by thin layer chromatography (TLC) on silica gel 60 F_254_ plates (Merck No 1.05715.0001: Darmstadt, Germany) developed with chloroform:methanol (3:1) to produce menthol glucoside and menthol galactoside with *R*_*f*_ values of 0.61 and 0.58, respectively, as detected with I_2_ vapor. Their structures were confirmed by 700 MHz Bruker, BioSpin nuclear magnetic resonance (NMR) analysis including one-dimensional ^1^H NMR, ^13^C NMR and two-dimensional NMR-correlation spectroscopy (COSY), hetero-nuclear single quantum coherence (HSQC), and heteronuclear multiple bond connectivity (HMBC).

### Water solubility determination

The water solubilities of (−)-menthol β-d-glucoside and (−)-menthol α-d-glucoside were tested. Each compound was dissolved at 20 mg/mL in distilled water. The solution was vortexed for 3 min, held overnight at 25 °C, and centrifuged at 6500*g* for 10 min. The concentration of each compound in the supernatants was quantitatively analyzed by LC–MS/MS analysis in MRM mode.

### Topical cooling test

Following approval of the study by the Public Institutional Review Board Designated by the Ministry of Health and Welfare (P01-201705-13-002), Korea, 10 healthy males and females were recruited from the local institute’s population.

The topical cooling test was performed as reported previously (Ottinger et al. 2001). Solutions of 1% (−)-menthol or (−)-menthol β-d-glucoside were prepared by first dissolving 100 mg of the product using 50 μL ethanol and diluting to 10 mL with water. Thus, the quantity of the alcoholic solution in the tested solutions was 0.5%. Twofold diluted solutions were prepared with 0.5% ethanol. An aliquot (0.2 mL) of solutions containing between 0.031 and 1.0% of the coolant in water was applied to a circular area (~10 cm^2^) of the skin surface on the inside of a forearm, midway between the wrist and the elbow, and rubbed for 30 s. In parallel, an aliquot (0.2 mL) of 0.5% ethanol was applied as a blank onto the skin of the other forearm. After 30 s, the skin was dried with a towel. A panel of 10 subjects was asked to identify the arm with a detectable “cooling” sensation and to rank the perceived cooling intensity on a scale from 0 (no effect) to 5 (very strong). The values evaluated in three different sessions over 2 days were averaged. The values between individuals and separate sessions differed by no more than two scores.

### In vitro skin sensitization test

The human cell line activation test (H-CLAT) using THP-1 (human monocytic cell line) was performed as described by Ashikaga et al. (2006). In brief, cells were cultured in RPMI 1640 medium (Invitrogen Corp., Carlsbad, CA, USA) with 10% FBS (v/v), 0.05 mM 2-mercaptoethanol, and 1% antibiotic–antimycotic mixture (Invitrogen Corp., Carlsbad, CA, USA), seeded at 0.5 × 10^6^ cells/mL in a 24-well plate, and cultured with chemicals for 24 h. When DMSO was used as a solvent, its final concentration in the culture media was less than 0.2%. The cell viability was evaluated by MTT assay. The concentration resulting in 75% cell viability, referred to as CV75, was calculated based on the analysis of viable cells. Cells were incubated for 24 h with test chemicals at three or four concentrations of a 1.2-fold serial dilution starting at 1.2 × CV75. The cells were analyzed for CD54 (with anti-CD54-FITC antibodies; DAKO, Denmark) and CD86 expression (with anti-CD86-FITC antibodies; BD Pharmingen) by flow cytometry. FITC labeled-mouse IgG1 was used as an isotype control. The relative fluorescence intensity (RFI) values of CD54 and CD86 were determined at >50% of cell viability and calculated as follows:

$$\frac{{\left( {{\text{MFI of chemical-treated cells}} - {\text{MFI of chemical-treated isotype control cells}}} \right)}}{{\left( {{\text{MFI of vehicle control cells}} - {\text{MFI of vehicle isotype control cells}}} \right)}}$$

where MFI is the mean fluorescence intensity. If the RFIs of CD54 and CD86 were greater than 200 and 150%, respectively, the test chemical was judged as a sensitizer, and otherwise, it was considered a non-sensitizer.

## Results

### Comparison of water solubility of (−)-menthol β-glucoside and (−)-menthol α-glucoside

**Fig. 1:**
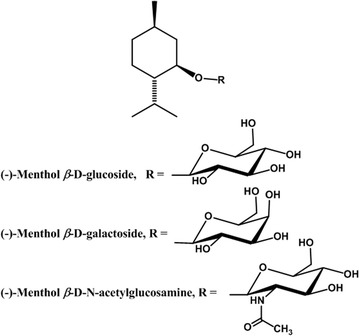
Structure of (−)-menthol β-d-glycosides synthesized by BLC, a glycosyltransferase from *B. licheniformis*

(−)-Menthol β-glucoside and (−)-menthol α-glucoside were prepared by chemical synthesis according to the a previously described method (Sakata and Iwamura 1979). The chemical synthesis produced an anomeric mixture, requiring tedious column chromatography to purify (−)-menthol β-glucoside. The water solubility values of (−)-menthol β-glucoside and (−)-menthol α-glucoside were compared and found to be remarkably different (Table 1). The solubility of (−)-menthol β-glucoside in water at 25 °C was 18.1 g/L, which is 27 times higher than that (0.66 g/L) of (−)-menthol α-glucoside. This result prompted us to conduct the enzymatic β-anomer-selective glycosylation of menthol (Fig. 1).

**Table 1:** Summary of water solubility and pharmacological properties of (−)-menthol β-d-glucoside and (−)-menthol

	Water solubility (g/L)	Flavor	The threshold concentration for cooling (%)	Expression of activation marker on THP-1 cells		Irritating sensation to the skin
				CD54	CD86	
Menthol	NT	Yes	0.125	Positive	Negative	Yes
Menthol β-glucoside	18.1	No	0.06	Negative	Negative	No
Control^a^	0.66	NT	NT	NT	NT	NT

*NT* not tested

^a^Control for water solubility were menthol α-glucoside

### Enzymatic glucosylation of menthol

**Fig. 2:**
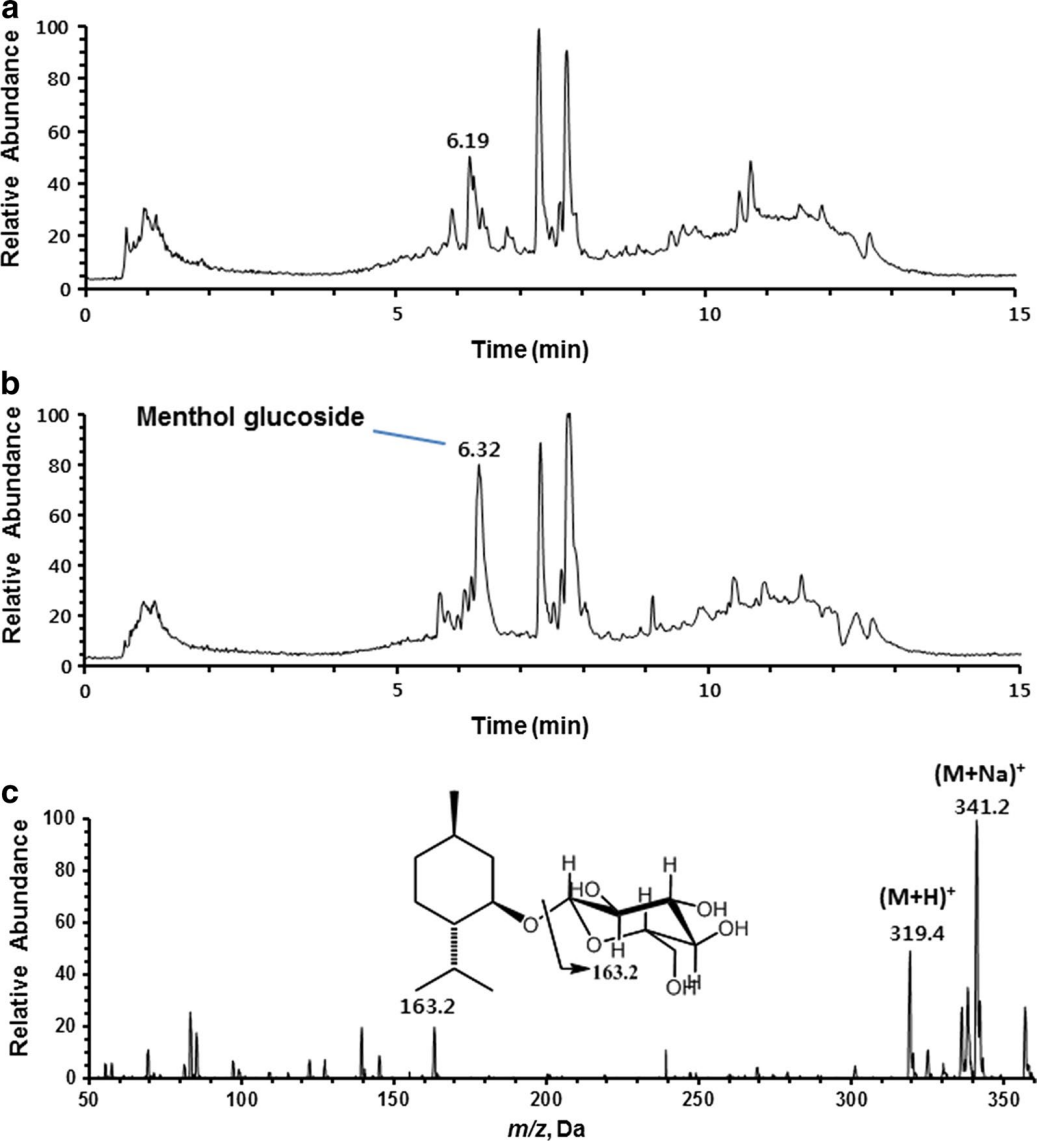
LC–MS analysis of (−)-menthol glycosylation reaction by BLC with UDP-d-glucose. **a** Total ion chromatogram of the reaction mixture at 0 h incubation (control reaction); **b** total ion chromatogram of the reaction mixture at 18 h incubation; **c** ESI–MS spectrum of the new peak at 6.32 min

**Fig. 3:**
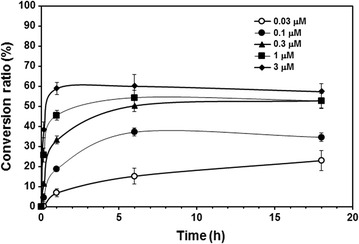
Time-course production of (−)-menthol β-d-glucoside using UDP-d-glucose as a sugar donor with increasing concentration of BLC (0.03–3 μM). The reaction mixtures were incubated for 10 min, 1, 6, and 18 h and quantitatively analyzed by LC–MS/MS in MRM mode according to “Materials and methods”. The *error bars* represent the standard deviation of three independent experiments

The purified BLC enzyme catalyzed the synthesis of (−)-menthol glucoside using UDP-d-glucose as a sugar donor (Fig. 2). Time-course production of (−)-menthol glucoside was quantitatively determined using LC–MS/MS in MRM mode (Fig. 3). BLC at 3 μM produced a maximum yield of nearly 58.9% in a molar ratio at 1 h. The *K*_*m*_ and *K*_*cat*_ for (−)-menthol with BLC were determined to be 1.74 ± 0.6 mM and 94.5 ± 2.33 min^−1^, respectively, in our system (Additional file 1: Figure S1).

### Menthol glycosylation with other UDP sugars

To examine the selectivity of BLC enzyme for sugar donors, menthol glycosylation was performed with other sugar donors, including UDP-d-galactose, UDP-*N*-acetylglucosamine, and UDP-d-glucuronic acid. Identical reactions were performed by varying only the UDP-sugar donor. The incubation of menthol with UDP-galactose and UDP-*N*-acetylglucosamine in the presence of BLC afforded new products corresponding to (−)-menthol galactoside and (−)-menthol *N*-acetylglucosamine, as confirmed by the LC–MS analysis (Additional file 1: Figures S2, S3), whereas no new product was detected with the use of UDP-glucuronic acid. The conversion of (−)-menthol to (−)-menthol galactoside was 1.56% in the large-scale preparation, as described in the Materials and Methods section. This result showed that BLC had a high selectivity for UDP-d-glucose as a sugar donor.

### Structure determination of menthol glycosides

After large-scale reactions followed by chromatographic isolation, (−)-menthol glucoside and (−)-menthol galactoside were obtained as white powders, and their structures were confirmed by HR-ESIMS and NMR spectral analysis (Additional file 1: Figures S4–S6). The anomeric configuration of (−)-menthol glucoside and (−)-menthol galactoside was determined to be β based on the large coupling constants of the anomeric protons at *δ* 4.35 (*J* = 7.8 Hz) and *δ* 4.35 (*J* = 7.6 Hz), respectively (Table 2). Because the yield of (−)-menthol *N*-acetylglucosamine was too low, the structure of (−)-menthol *N*-acetylglucosamine was determined using only MS/MS and HR-ESI MS data (*m/z* 382.3 [M+Na]^+^, 360.3 [M+H]^+^, 204.2 [*N*-acetylglucosamine+H] ^+^; HRESIMS: *m/z* 360.2385 (M+H)^+^, calcd. 360.2381 for C_18_H_34_O_6_N). To the best of our knowledge, the NMR data of menthol β-d-glucoside was reported for the first time in this study, and (−)-menthol β-d-galactoside and (−)-menthol *N*-acetylglucosamine are novel compounds.

**Table 2:** ^1^H- and ^13^C-NMR data (700 MHz, CD_3_OD) for biosynthesized (−)-menthol β-glucoside and (−)-menthol β-galactoside

Position	(−)-Menthol β-glucoside		(−)-Menthol β-galactoside	
	δ_H_ (multi., J in Hz)	δ_c_	δ_H_ (multi., J in Hz)	δ_c_
1	3.57 (1H, ddd, 10.6, 105, 4.2)	78.4	3.57 (1H, ddd, 10.8, 10.8, 4.4)	78.4
2	1.24 (1H, m)	49.5	1.22 (1H, m)	49.5
3	1.03 (1H, m); 1.63 (1H, m)	24.3	1.03 (1H, m); 1.65 (1H, m)	24.4
4	0.84 (1H, m); 1.68 (1H, m)	35.8	0.85 (1H, m); 1.67 (1H, m)	35.8
5	1.37 (1H, m)	32.9	1.38 (1H, m)	32.9
6	0.94 (1H, m); 2.12 (1H, m)	41.8	0.93 (1H, m); 2.13 (1H, m)	41.8
7	2.31 (1H, m)	26.3	2.31 (1H, m)	26.3
8	0.81 (3H, d, 6.8)	16.4	0.79 (3H, d, 7.2)	16.4
9	0.87 (3H, d, 7.1)	21.6	0.89 (3H, d, 7.0)	21.6
10	0.93 (3H, d, 6.6)	22.8	0.96 (3H, d, 6.8)	22.8
1′	4.35 (1H, d, 7.8)	101.4	4.35 (1H, d, 7.6)	101.5
2′	3.14 (1H, m)	75.2	3.14 (1H, m)	75.2
3′	3.35 (1H, m)	78.3	3.35 (1H, m)	78.3
4′	3.31 (1H, m)	72.0	3.30 (1H, m)	72.1
5′	3.24 (1H, m)	77.8	3.24 (1H, m)	77.8
6′	3.67 (1H, dd, 11.6, 5.4); 3.84 (1H, dd, 11.6, 2.3)	63.1	3.67 (1H, dd, 11.6, 5.2); 3.84 (1H, dd, 11.6, 2.4)	63.2

### Cooling effect

**Fig. 4:**
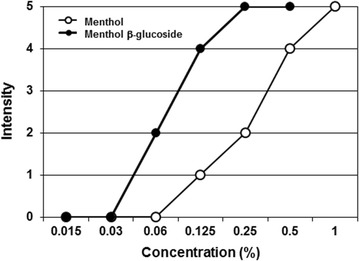
Topical testing of (−)-menthol β-d-glucoside and (−)-menthol on the inside of the forearm. The cooling effect on the skin was determined using a topical test that scored the cooling intensity on a scale from 0 (no effect) to 5 (very strong)

To determine whether glucosylation affects the cooling sensation of menthol on the skin, topical cooling tests of (−)-menthol β-d-glucoside were performed using menthol as the control. (−)-Menthol β-d-glucoside showed a higher cooling effect than menthol (Fig. 4; Additional file 1: Figure S7) and was topically detectable at a concentration of 0.06%, whereas menthol imparted a cooling sensation at a concentrations of >0.125%. Increasing the concentration of (−)-menthol β-d-glucoside intensified the cooling effect on the skin, reaching the maximum score of 5 at a concentration of 0.25%, whereas menthol required a concentration of 1%.

### Skin sensitizing potential

**Fig. 5:**
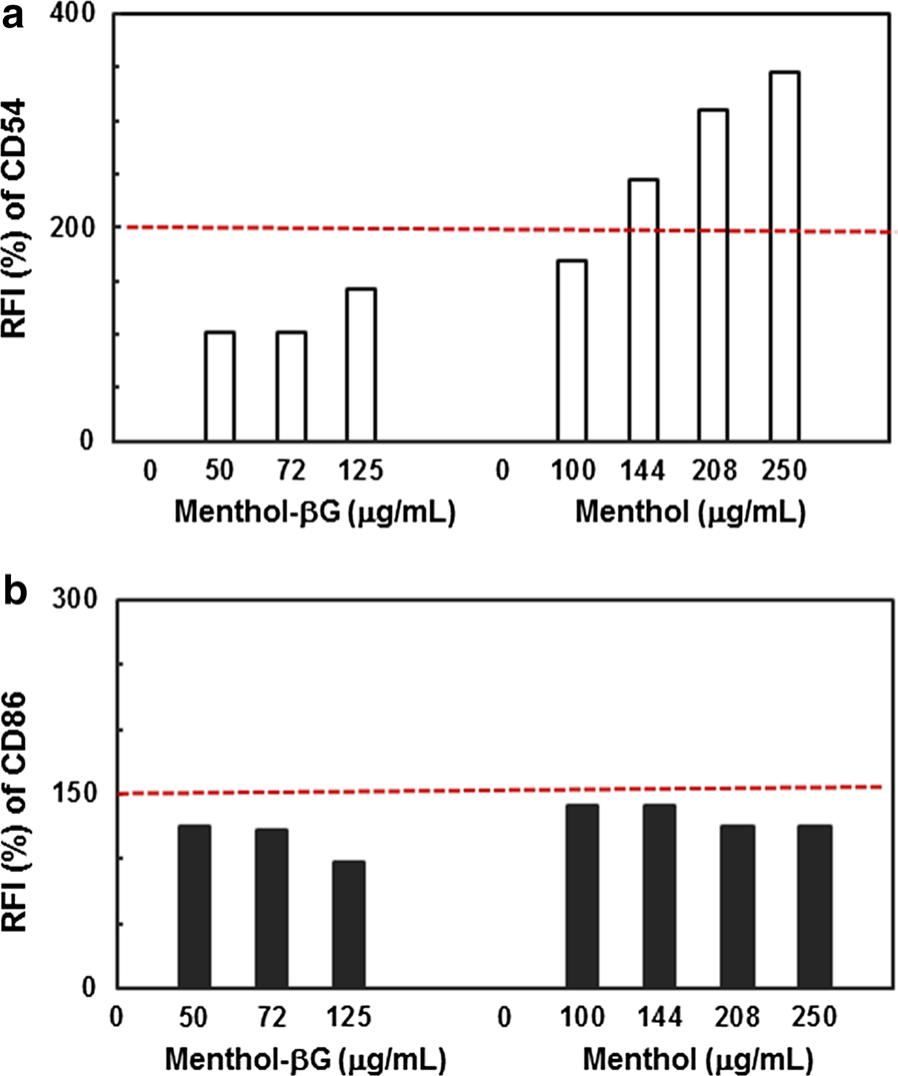
In vitro skin sensitization test of (−)-menthol β-d-glucoside and (−)-menthol using human cell lines. The changes in CD54 (**a**) and CD86 (**b**) expression on THP-1 cells were measured after a 24-h exposure to a test compound. The RFI values were calculated and compared

The in vitro skin sensitization test was performed to determine the sensitizing potential by assessing the changes in CD54 and CD86 expression in THP-1 cells using flow cytometry after 24 h exposure to a test compound (Fig. 5). Menthol and (−)-menthol β-glucoside treatments were applied at three or four concentrations of a 1.2-fold serial dilution starting at 250 and 125 μg/mL, respectively, which were the concentrations that resulted in 75% cell viability. As expected, menthol presented values >200% RFI for CD54 activation marker, indicating the sensitizing potential of menthol. However, (−)-menthol β-glucoside presented values <150% RFI for both CD54 and CD86 activation markers, indicating that (−)-menthol β-glucoside is a non-sensitizer. In fact, the sensory panel did not feel any significant irritating sensation to the skin for (−)-menthol β-d-glucoside during the topical cooling tests, whereas menthol did produce an irritating sensation.

## Discussion

Menthol has well-known cooling characteristics and a residual minty smell. Because of these attributes, menthol is used in a variety of consumer products, ranging from confectioneries such as chocolate and chewing gum to cosmetics, oral-care products such as toothpaste, in over-the-counter medicinal products such as analgesics, and as an additive in cold compresses for its cooling and biological effects (Eccles 1994; Patel et al. 2007). Additionally, approximately one-quarter of the cigarettes on the market contain menthol (Kamatou et al. 2013). However, due to its poor solubility in water, the use of menthol has been limited to direct mixing in the form of solid particles with the material to which the menthol is to be added, making a suspension using an emulsifier, or dissolving the menthol in an organic solvent such as alcohol. Additionally, the inherent mint flavor limits the use of menthol in certain cooling products, such as cosmetics. Thus, menthol derivatives with good solubility in water and no flavor that retain the cooling sensation have been greatly desired. Glycosylation improves the water solubility of drugs or natural products (Weymouth-Wilson 1997). In our study, (−)-menthol β-glucoside was shown to more soluble in water (>27 times) than (−)-menthol α-glucoside; hence, the β-anomer-selective glucosylation of menthol is necessary. Limited glycosylation of menthol has been reported. Water-soluble menthyl glycosides with monosaccharide or oligosaccharide as a sugar unit have been synthesized by chemical methods, but the anomer-selective synthesis of methyl glycoside is impossible due to a lack of regioselectivity and stereoselectivity (Sakata and Iwamura 1979). α-Anomer-selective synthesis of menthyl glucoside was reported using α-glucosidase from yeast (Nakagawa et al. 1998) or bacteria such as Xanthomonas (Nakagawa et al. 2000), but β-anomer-selective glucosylation of menthol has not yet been reported. We therefore undertook to synthesize menthol β-glucoside by synthesizing the glucosyl analogs of menthol using UDP-glycosyltransferase BLC from *B. licheniformis*. Three different glycoside derivatives with the β-configuration were successfully produced in the reaction catalyzed by the BLC in the presence of the corresponding NDP-sugars. The maximum glucoside conversion rate was determined from the time-dependent study of menthol coupled with UDP-d-glucose. During the molar conversion, approximately 58.9% glucoside was produced after 1 h of incubation. BLC from *B. licheniformis* DSM 13 is reported to glycosylate diverse substrates such as geldanamycin analogs (Wu et al. 2012), epothilone A (Parajuli et al. 2014), chalcone (Pandey et al. 2013), and various flavonoids (Koirala et al. 2014). The maximum conversion rates of epothilone A and 3-hydroxyflavone to its respective glucosides by BLC were reported to be approximately 26 and 90%, respectively, at 3 h incubation using UDP-d-glucose. In this study, BLC catalyzed the glycosylation of menthol (C_10_H_20_O; molecular weight, 156), a notably small cyclic monoterpene alcohol. This result proves the remarkable flexibility of an aglycone substrate of BLC.

Although improvement of the water solubility of menthyl glycosides is known (Sakata and Iwamura 1979), no reports exist regarding the enhancement of their pharmacological properties. Menthol acts on the highly sensitive cold receptor TRPM8 located on the cell membrane of sensory neurons (McKemy et al. 2002). Menthol contains many active forms, but (−)-menthol produces the strongest cooling effects. Low concentrations of (−)-menthol elicit a cooling sensation (Schafer et al. 1986), but increased concentrations result in irritation and local anesthetic effects (Eccles 1994). The feeling of irritation might be due to the action of (−)-menthol on pain fibers (Eccles 1994). A concentration of 0.2% (−)-menthol causes both cooling and irritation. Thus, an optimal concentration of menthol that maintains a cooling sensation and minimizes irritation must be considered (Gillis et al. 2010). In this study, interestingly, (−)-menthol β-d-glucoside exhibited higher cooling effects with no accompanying irritation compared with menthol. We believe that the sugar moiety of (−)-menthol β-d-glucoside might play an important role in its interaction with the cold receptor and pain fibers.

Many natural products currently used as therapeutics derive their biological functions or pharmacokinetic properties from the sugar components present in their structures. For example, vancomycin loses its in vivo efficacy if the sugar moiety is removed, although in vitro activity remains (Nagarajan 1993). Doxorubicin has no antitumor activity without the sugar portion (Han et al. 2011). Erythromycin contains sugar moieties that affect its molecular mechanism of action (Langenhan et al. 2005). Different sugar moieties could also result in alteration of biological activities, as observed in rebeccamycin (Animati et al. 2008). In addition to the cooling effect, menthol has diverse biological properties such as analgesic, antifungal, antibacterial, anticancer, anti-inflammatory, antitussive, antiviral, and insecticidal effects (Kamatou et al. 2013). In recent years, menthol has become one of the most effective terpenes used to enhance the dermal penetration of pharmaceuticals. Thus, a study on the effects of menthol glycosides on these biological activities is of interest.

The current study demonstrates a higher water solubility, an improvement in cooling effects and a lack of sensitization of (−)-menthol β-glucoside, thereby demonstrating its potential as a new cooling agent. β-Anomer-selective glucosylation of (−)-menthol was achieved using the BLC glycosyltransferase from *B. licheniformis*. Because BLC uses the relatively expensive UDP-glucose as a donor substrate, the in vivo production of (−)-menthol β-glucoside using a strain engineered with BLC could be considered for industrial production of menthol β-glucoside.

## Additional file

**Additional file 1.** Structural elucidation, LC–MS and HR-ESIMS data of (−)-menthol β-glucoside, (−)-menthol β-galactoside, and (−)-menthol *N*-acetylglucosamine. Lineweaver–Burk plot of (−)-menthol glucosylation by BLC with respect to menthol (**Figure S1**). LC–MS analysis of (−)-menthol glycosylation reaction by BLC with UDP-d-galactose (**Figure S2**) and UDP-d-*N*-acetylglucosamine (**Figure S3**). ^1^H NMR spectra of synthesized (−)-menthol α-glucoside and (−)-menthol β-glucoside (**Figure S4**). ^1^H and ^13^C NMR spectra of (−)-menthol β-glucoside (**Figure S5**) and (−)-menthol β-galactoside (**Figure S6**) prepared by BLC-catalyzed glycosylation reactions. HMBC data of the menthol glucoside and menthol galactoside prepared by BLC-catalyzed glycosylation reactions (**Figure S7**). Topical cooling test (**Table S1**).

## Abbreviations

UDP: uridine diphosphate
MRM: multiple reaction monitoring
TLC: thin layer chromatography
NMR: nuclear magnetic resonance
H-CLAT: human cell line activation test
THP-1: human monocytic cell line
RFI: relative fluorescence intensity
