# Supplementary material for: Improvement of the pharmacological activity of menthol via enzymatic β-anomer-selective glycosylation
Source: AMB Express. 2017 Aug 29;7:167. doi: 10.1186/s13568-017-0468-0 (PMC5574827; doi:10.1186/s13568-017-0468-0)
Supplement: Supplementary file 1 — Additional file 1. Structural elucidation, LC–MS and HR-ESIMS data of (−)-menthol β-glucoside, (−)-menthol β-galactoside, and (−)-menthol N-acetylglucosamine. Lineweaver–Burk plot of (−)-menthol glucosylation by BLC with respect to menthol (Figure S1). LC–MS analysis of (−)-menthol glycosylation reaction by BLC with UDP-d-galactose (Figure S2) and UDP-d-N-acetylglucosamine (Figure S3). 1H NMR spectra of synthesized (−)-menthol α-glucoside and (−)-menthol β-glucoside (Figure S4). 1H and 13C NMR spectra of (−)-menthol β-glucoside (Figure S5) and (−)-menthol β-galactoside (Figure S6) prepared by BLC-catalyzed glycosylation reactions. HMBC data of the menthol glucoside and menthol galactoside prepared by BLC-catalyzed glycosylation reactions (Figure S7). Topical cooling test (Table S1). [file 13568_2017_468_MOESM1_ESM.pdf]

Structural elucidation of menthol glycosides

The ^{1}H- and ^{13}C-NMR spectroscopic data for purified menthol glucoside together with two-dimensional (2D) NMR including ^{1}H-^{1}H COSY, HMQC and HMBC were measured. The ^{1}H-NMR spectrum showed the presence of an anomeric proton at the chemical shift δ 4.35 (1H, d, J = 7.8 Hz, H-1’), indicating an anomeric proton with a β configuration of the sugar moiety. The glucosylation position was determined by HMBC analysis (Fig. S6A). The anomeric proton at δ 4.35 was long-range coupled to the oxygenated methine carbon at δ 78.4 (C-1) of the aglycon. Thus, together with HR-ESIMS data, the product was determined as (-)-menthol β–D-glucoside.

The chemical structure of the purified menthol galactoside was also determined in the same manner. The anomeric proton at the chemical shift δ 4.35 (1H, d, J = 7.6 Hz, H-1’), indicating an anomeric proton with a β configuration of the sugar moiety, has HMBC correlation with the oxygenated methine carbon at δ 78.4 (C-1) of the aglycon in HMBC spectrum (Fig. S6B). The other portion was confirmed by the HMBC. Thus, together with HR-ESIMS data, menthol galactoside was determined as a new (-)-menthol β–D-galactoside.

Because the yield of (-)-menthol N-acetylglucosamine was too low, the structure of (-)-menthol N-acetylglucosamine was determined using only MS/MS and HR-ESI data.

LR-ESIMS and HR-ESIMS data of menthol glycosides

Menthol β–D-glucoside. A white powder; ESI-MS : 341.2 $[M+Na]^+$, 319.4 $[M+H]^+$, 163.2 $[glucose+H]^+$; HR-ESIMS: *m/z* 319.2117 $(M+H)^+$, $C_{16}H_{31}O_6$ requires 319.2115; *m/z* 341.1936 $(M+Na)^+$, $C_{16}H_{30}O_6Na$ requires 341.1940.

Menthol β–D-galactoside. A white powder; ESI-MS : 341.3 $[M+Na]^+$, 319.3 $[M+H]^+$, 163.3 $[glucose+H]^+$; HR-ESIMS: *m/z* 319.2117 $(M+H)^+$, $C_{16}H_{31}O_6$ requires 319.2115; *m/z* 341.1934 $(M+Na)^+$, $C_{16}H_{30}O_6Na$ requires 341.1940.

Menthol β–D-N-acetylglucosamine. A white powder; ESI-MS : 382.3 $[M+Na]^+$, 360.3 $[M+H]^+$, 204.2 $[N\text{-acetylglucosamine}+H]^+$; HR-ESIMS: *m/z* 360.2385 $(M+H)^+$, $C_{18}H_{34}O_6N$ requires 360.2381; *m/z* 382.2201 $(M+Na)^+$, $C_{18}H_{33}O_6NNa$ requires 382.2205.

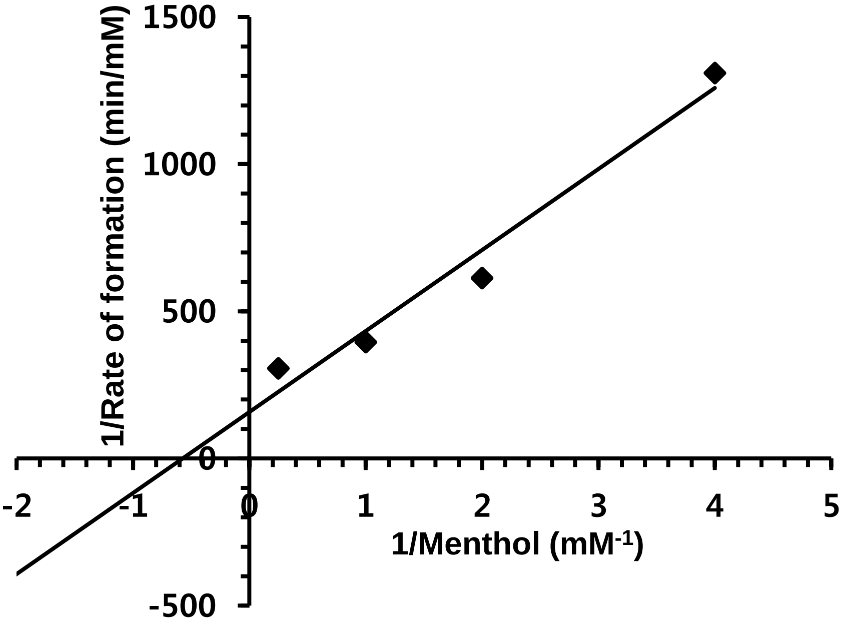

Fig S1. Lineweaver-Burk plot of (-)-menthol glucosylation by BLC respective to menthol.

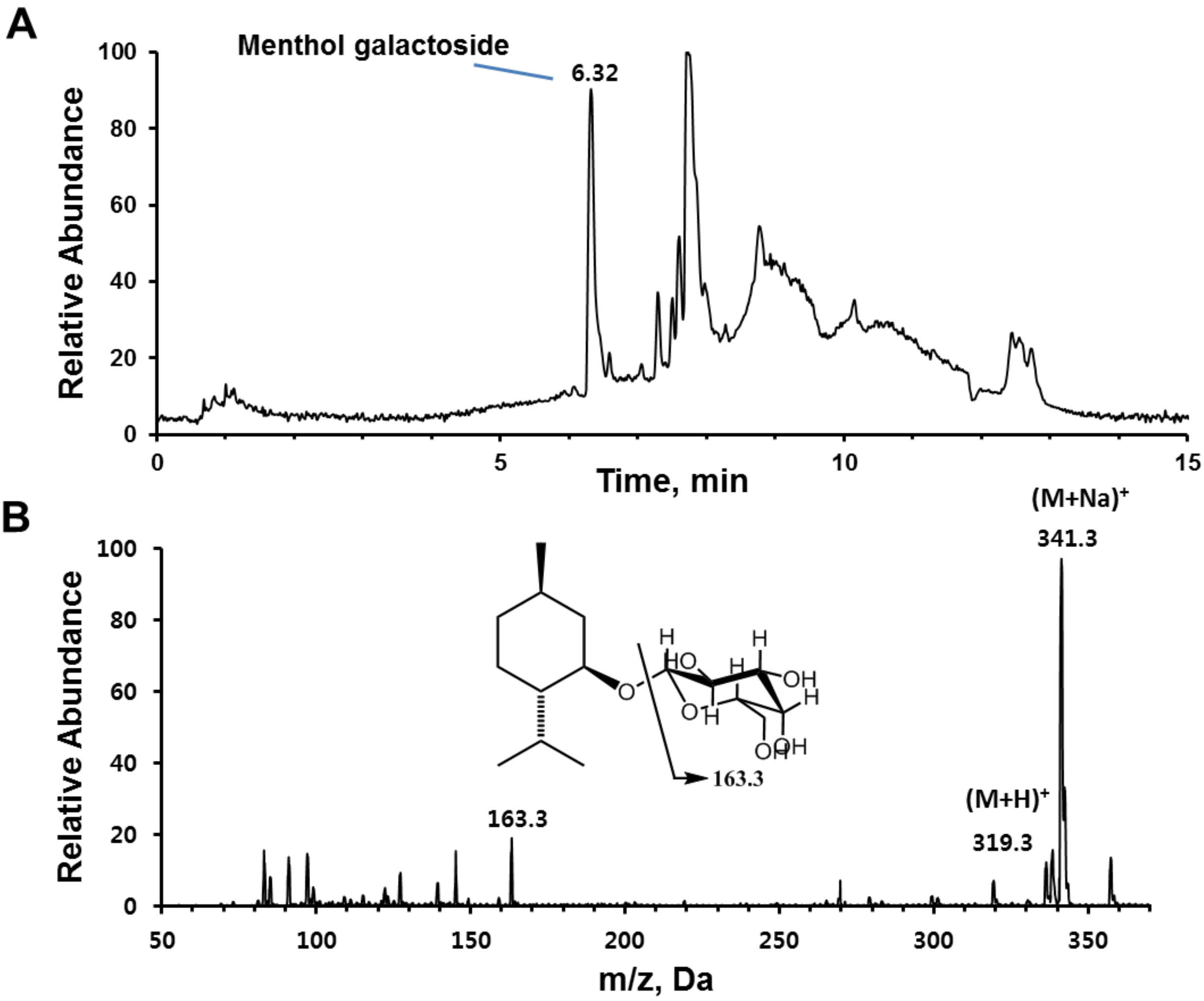

Fig. S2. LC-MS analysis of (-)-menthol glycosylation reaction by BLC with UDP-D-galactose. A, total ion chromatogram of the reaction mixture; B, ESI-MS spectrum of the new peak at 6.32 min.

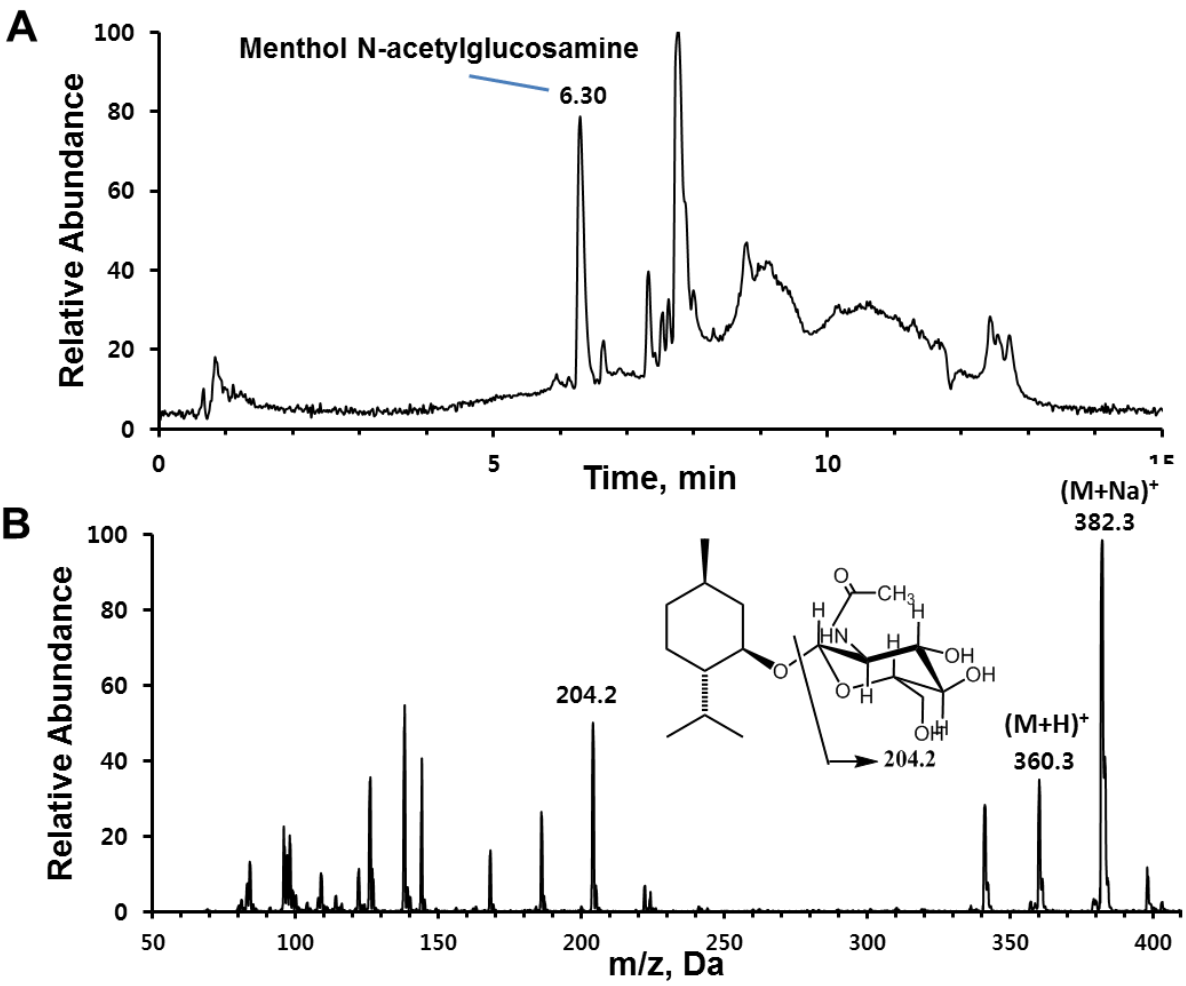

Fig. S3. LC-MS analysis of (-)-menthol glycosylation reaction by BLC with UDP-D-N-acetylglucosamine. A, total ion chromatogram of the reaction mixture; B, ESI-MS spectrum of the new peak at 6.30 min.

A

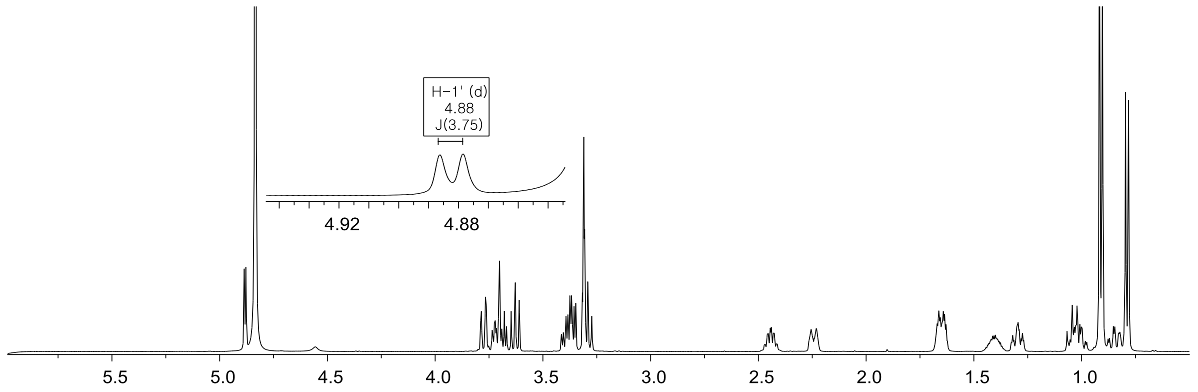

B

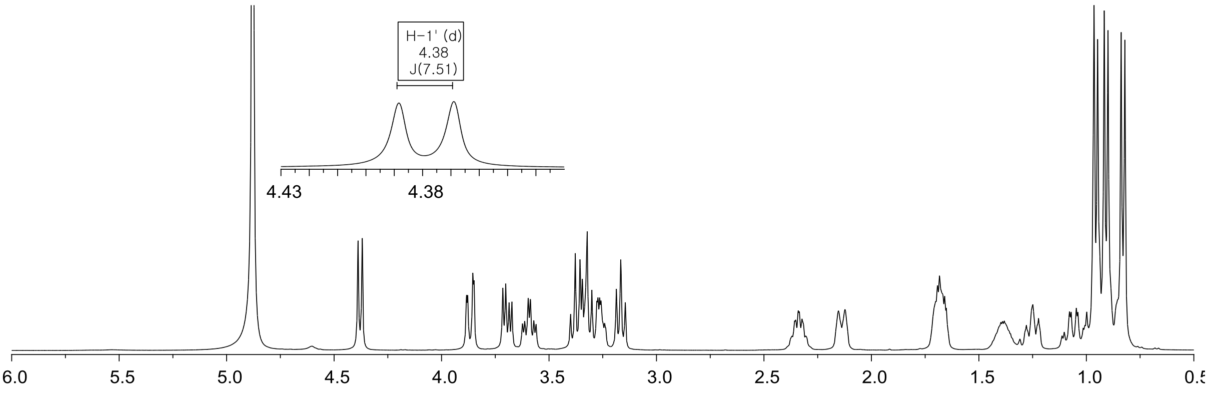

Fig. S4. ^{1}H NMR spectra of synthesized (-)-menthol α-glucoside (A) and (-)-menthol β-glucoside (B) measured at 500 MHz in CD_3OD.

A

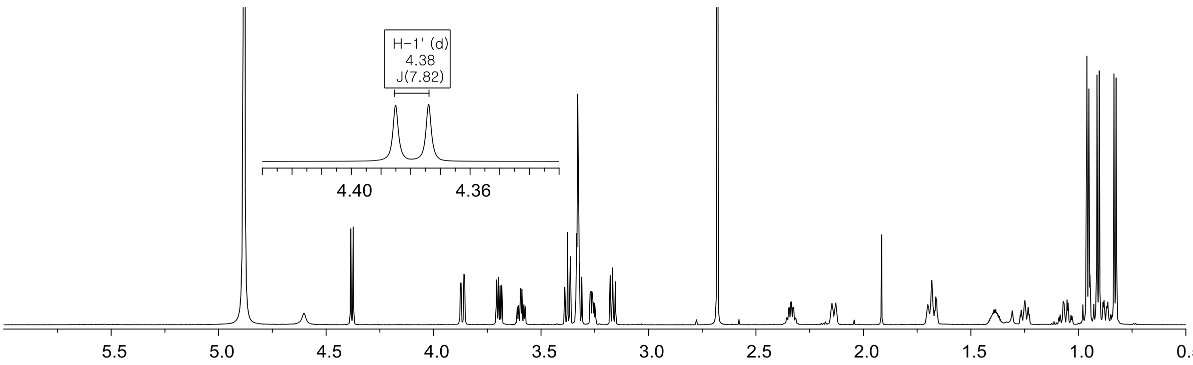

B

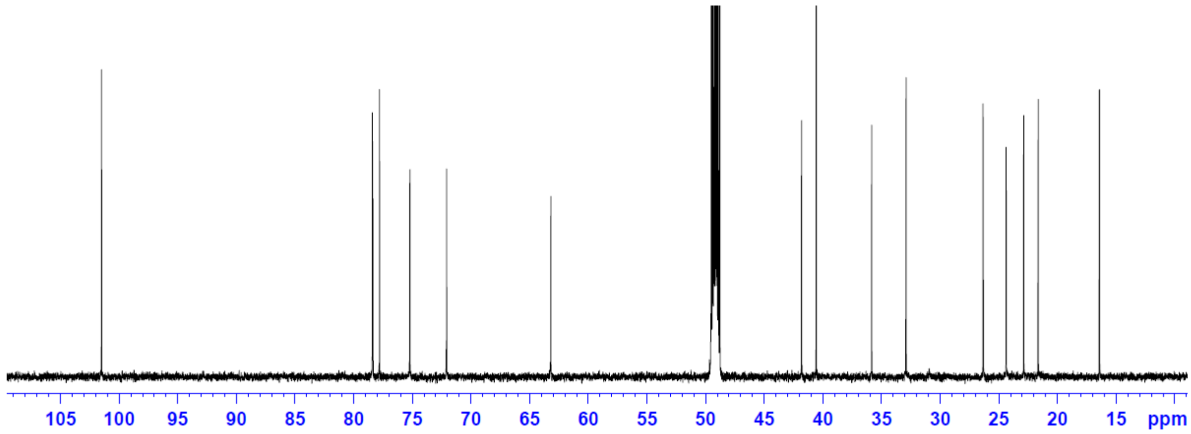

Fig. S5. ^{1}H and ^{13}C NMR spectra of (-)-menthol β-glucoside (B and C) prepared by BLC-catalyzed glycosylation reactions. ^{1}H (A) and ^{13}C NMR (B) were measured at 700 and 175 MHz, respectively, in CD_3OD.

A

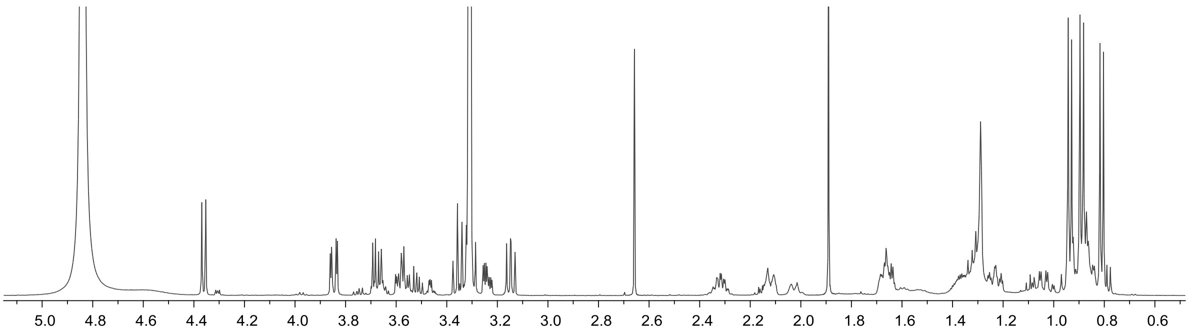

B

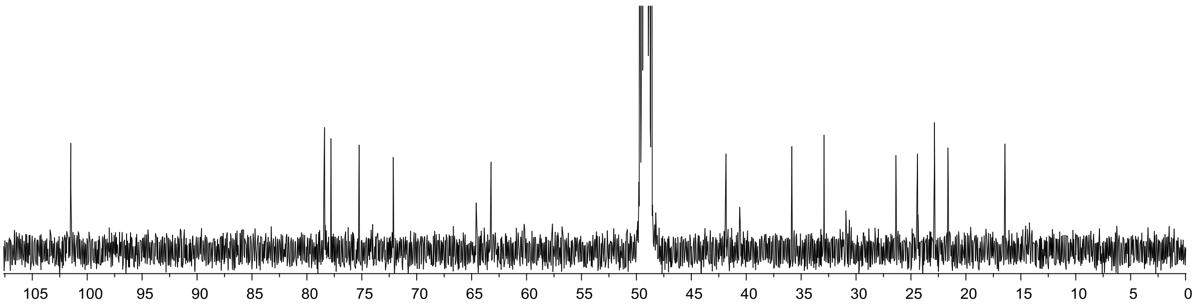

Fig. S6. ^{1}H and ^{13}C NMR spectra of (-)-menthol β-galactoside prepared by BLC-catalyzed glycosylation reactions. ^{1}H (A) and ^{13}C NMR (B) were measured at 700 and 175 MHz, respectively, in CD_3OD.

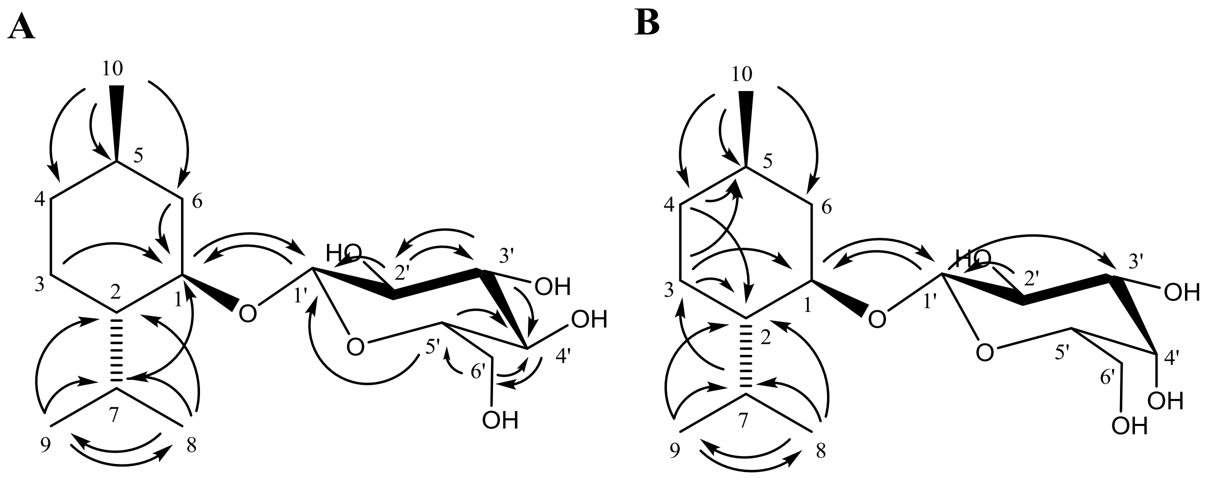

Fig. S7. HMBC data of the menthol glucoside (**A**) and menthol galactoside (**B**) prepared by BLC-catalyzed glycosylation reactions.

Table S1. Topical cooling test

Menthol (%)	#1	#2	#3	#4	#5	#6	#7	#8	#9	#10	Sum	Average
0.0625	0	0	0	0	0	0	0	0	1	0	1	0.1
0.125	0	1	1	0	1	2	1	1	2	1	10	1
0.25	1	1	3	1	3	2	2	2	3	3	21	2.1
0.5	4	3	4	3	4	4	4	4	5	4	39	3.9
1	5	5	5	5	5	5	5	5	5	5	50	5

Menthol β-glucoside (%)	#1	#2	#3	#4	#5	#6	#7	#8	#9	#10	Sum	Average
0.0312	1	0	0	0	0	0	0	0	0	0	1	0.1
0.0625	3	2	2	2	2	2	2	2	2	1	20	2
0.125	5	4	4	3	4	4	3	4	4	4	39	3.9
0.25	5	5	5	5	5	5	5	5	5	5	50	5
0.5	5	5	5	5	5	5	5	5	5	5	50	5

The perceived cooling intensity was scored on a scale from 0 (no effect) to 5 (very strong).
